# Effect of tyrosine-rich amelogenin peptide on behavior and differentiation of endothelial cells

**DOI:** 10.1007/s00784-016-1726-2

**Published:** 2016-02-12

**Authors:** Erwin Jonke, Anja C. Gemperli, Taowen Zhang, Burcu Özdemir, Michel Dard, Xiaohui Rausch-Fan, Oleh Andrukhov

**Affiliations:** 1 Division of Orthodontics, University Clinic of Dentistry, Medical University of Vienna, Vienna, Austria; 2 Institut Straumann AG, Basel, Switzerland; 3 Division of Conservative Dentistry and Periodontology, University Clinic of Dentistry, Medical University of Vienna, Vienna, Austria; 4 Yantai Stomatological Hospital, Binzhou Medical University, Yantai, China; 5 Department of Periodontology, Faculty of Dentistry, Gazi University, Ankara, Turkey; 6 College of Dentistry, Department of Periodontology and Implant Dentistry, New York University, New York, NY USA

**Keywords:** Enamel matrix derivative, Wound healing, Endothelial cells, Angiogenesis

## Abstract

**Background:**

Enamel matrix derivative (EMD) is an effective biomaterial for periodontal tissue regeneration and might stimulate angiogenesis. Tyrosine-rich amelogenin peptide (TRAP) is present in EMD and is thought to contribute in its biological activity. In the present study, we investigated the effect of chemically synthesized TRAP on proliferation, migration, angiogenic structure formation, and differentiation of human umbilical vein endothelial cells (HUVECs) in vitro.

**Material and methods:**

The effects of TRAP isolated from EMD and chemically synthesized TRAP on proliferation/viability, migration, and angiogenic structure formation were investigated. Expression of angiopoietin-2 (ang-2), von Willebrand factor (vWF), E-selectin, intracellular adhesion molecules 1 (ICAM-1), vascular endothelial growth factor (VEGF) receptors FMS-like tyrosine kinase 1 (FLT-1), and kinase insert domain receptor (KDR) was measured on both messenger RNA (mRNA) and protein levels.

**Results:**

The proliferation/viability of HUVECs was inhibited by TRAP at concentration of 100 μg/ml and slightly stimulated by EMD at similar concentration. Both EMD and TRAP stimulated endothelial cell migration in microchemotaxis chamber. The effect of both TRAP preparations on the migration was significantly higher than that of EMD. All substances stimulated formation of angiogenic structure in vitro. The expression of ICAM-1, E-selectin, FLT-1, KDR, and vWF was significantly increased by both TRAP and EMD at a concentration 50 μg/ml. The expression of ang-2 was not affected by TRAP but was significantly increased by EMD.

**Conclusion:**

Our in vitro study shows that TRAP confer the most effects of EMD on the endothelial cells.

**Clinical relevance:**

TRAP might be used as a basis for development of new approaches for periodontal regeneration.

## Introduction

Application of bioactive material is considered an important approach to improve the regeneration of periodontal tissue. Enamel matrix derivative (EMD) is a complex of low molecular weight hydrophobic enamel proteins, which is derived from developing porcine tooth buds. The EMD-based commercial product Emdogain, which contains also a propylene glycol alginate (PGA) carrier, has been used clinically since more than 10 years, and its capacity to promote periodontal regeneration has been largely documented [1, 2]. The biological effects of EMD are probably due to the presence of bioactive compounds, which are involved in the process of teeth development [3, 4].

Periodontium is highly vascularized tissue and therefore success of therapy depends on the ability to promote the formation of blood microvessels, which guarantee nutrition and oxygen supply. Several in vitro and in vivo studies show that EMD stimulates angiogenesis [5, 6], a process of new vessels formation playing an important role in periodontal regeneration and wound healing [7]. Endothelial cells (ECs), which underlie the inner surface of the vasculature, play a key role in the angiogenesis. The process of new vessel formation includes sprouting of ECs from the existing vessel, proliferation, migration, and organization in the capillary network [8]. Several in vitro studies show that EMD stimulates migration, angiogenesis, and expression of angiogenesis-related proteins in ECs [9–12]. Moreover, EMD is recently shown to stimulate angiogenic differentiation of periodontal ligament-derived stem cells [13].

EMD proteins responsible for its biological activity are not exactly identified. EMD is composed mainly from amelogenins accounting about 90 % of all proteins [3]. Amelogenins are the family of proteins and low molecular weight peptides derived from single gene and formed by alternative splicing and proteolytic degradation. Besides amelogenin proteins, EMD contains also ameloblastin, enamelin, tuftelin, and proteolytic enzyme [3]. The mechanism underlying the regenerative ability of EMD as well as an exact bioactive EMD compound(s) is a matter of debate. Recently, a model of EMD action in vivo was proposed [3]. According to this model, EMD forms in vivo a multilayer composed of amelogenin nanospheres, which entraps bioactive EMD components. Upon contact with aqueous solution, bioactive compounds are released from EMD nanospheres.

Some previous study attempted to identify proteins responsible for the angiogenic activity of EMD. Besides whole length amelogenin, an angiogenic activity was observed for EMD-derived peptide with molecular weight of about 5 kDa, which is presumably a tyrosine-rich amelogenin peptide (TRAP). TRAP represents N-terminus of 20 kDa amelogenin and is a product of its proteolytic degradation [14]. A recent study show that chemically synthesized TRAP stimulates angiogenic differentiation of human periodontal ligament stem cells [15]. However, the effect of TRAP on endothelial cells, which play a key role in angiogenesis, is not investigated to date. Therefore, in the present study, we investigated the effect of TRAP on proliferation/viability, migration, and differentiation of human umbilical vein endothelial cells in vitro. Two different TRAP preparations were used: TRAP isolated from EMD and synthetic TRAP.

## Material and methods

### Cells and materials

Commercially available human umbilical vein endothelial cells (HUVECs) pooled from 10 different healthy donors (Technoclone, Vienna, Austria) were used in the present study. HUVECs were cultured in endothelial cell medium (ECM, Technoclone, Austria) with 20 % fetal bovine serum (FBS) supplemented with 100 U/ml penicillin, 100 μg/ml streptomycin, 0.25 μg/ml fungizone, 2 mM l-glutamine, 5 U/ml heparin, and 30–50 μg/ml endothelial cell growth supplement in culture flasks coated with 0.2 % gelatine at 37 °C in a humidified atmosphere of 5 % CO_2_ and 95 % air. The HUVECs from the 3rd to 6th passage in culture were used.

Two different TRAP preparations were used in the present study. First, TRAP was separated and purified from EMD by Institut Straumann using a modification of previously described methods [16, 17]. This preparation consisted of TRAP peptides with either 43 or 45 amino acid residues (e-TRAP). Second, synthetic TRAP peptide was produced by Straumann Institute. Lyophilized substances were reconstituted in 0.1 % acetic acid to produce a 10 mg/ml stock solution. Further dilutions of proteins (1–100 μg/ml) were performed into FBS-free ECM. In each experiment, ECM supplemented with the acetic acid at concentrations of 0.0001–001 % was used as a vehicle control. No significant effect of acetic acid on any study parameter was observed.

### Cell proliferation/viability

Cell proliferation/viability was measured using 3,4,5-dimethylthiazol-2-yl-2,5-diphenyl tetrazolium bromide (MTT) dye [18]. HUVECs were seeded in 24-well plates coated with 0.2 % gelatine at a density of 2 × 10^4^ cells per well in 0.5 ml of ECM supplemented with 20 % FBS. After 24 h, the medium in test wells was replaced by FBS-free ECM conditioned with e-TRAP, synthetic TRAP, or EMD at concentrations of 1–100 μg/ml. Wells, stimulated with FBS-free ECM supplemented with 0.001 % of acetic acid served as vehicle controls. After 24 h incubation, 100 μl of MTT solution (5 mg/ml in PBS) were added into each well and culture plates were incubated at 37 °C for 4 h. The medium was removed and 500 μl dimethylsulfoxide (DMSO) were added to each well, followed by 5 min incubation on a shaker. Finally, 100 μl of each cultured solution were transferred to a separate 96-well plate and the optical density (OD) was measured at 570 nm with an ELISA Reader (Molecular Devices, USA).

### Chemotaxis assay

Cell migration was assessed in a 48-well microchemotaxis chamber (Neuroprobe, Gaithersburg, MD, USA) as described previously [19]. The chamber consisted of acrylic top and bottom plates, each containing 48 matched wells. Twenty-six microliters of FBS-free medium containing tested substance (10 μg/ml) were filled in wells of the bottom plate. Wells filled with medium containing 0.0001 % of acetic acid served as control. Subsequently, the bottom plate was covered with a polycarbonate filter with 8-μm pore size (Neuroprobe, Gaithersburg, MD, USA) and the top plate was applied so that each well corresponded to that of the bottom plate. 1 × 10^4^ cells resuspended in 50 μl FBS-free medium were added to each well of the top plate and the whole chamber was incubated at 37 °C in humidified air with 5 % CO_2_ for 8 h. After incubation, cells on the upper surface of the filter were removed over the wiper blade and the filters were then fixed with methanol and stained using Hemacolor staining kit (Merck, Darmstadt, Germany). The cells migrated across the filter were counted under a light microscope at high-power magnification (×100) to measure transmigration in each well. Four fields were counted in each well and the total number was calculated. Four wells were used for each group; experiments were repeated in triplicate.

### Formation of angiogenic structure in vitro

The formation of angiogenic structures in vitro was performed using angiogenesis assay kit (Life Technologies, Grand Island, NY, USA) according to manufacturer’s instruction. 5 × 10^4^ cells were seeded in a 4-well plate precoated with Geltrex Matrix in 0.5 ml of medium 200 supplemented with large vessel endothelial supplement (all from Life Technologies). Cells were cultured in the presence of e-TRAP, synthetic TRAP, or EMD at a concentration of 10 μg/ml. Cells cultures in the presence of 0.0001 % of acetic acid were used as a vehicle control. After 15 h, digital images were obtained using light microscope (Nikon Eclipse TS100) with mounted digital camera.

### Measurements of gene expression levels by quantitative real-time PCR

Messenger RNA (mRNA) expression levels of E-selectin, intracellular adhesion molecules (ICAM-1), FMS-like tyrosine kinase 1 (FLT-1), kinase insert domain receptor (KDR), angiopoietin-2 (ang-2), and von Willebrand factor (vWF) were determined by qPCR similarly to the method described in our previous studies [20–22]. Glyceraldehyde-3-phosphate dehydrogenase (GAPDH) was used as a house-keeping gene. HUVECs were seeded in 24-well plates similar to MTT experiments and stimulated in FBS-free ECM with e-TRAP, synthetic TRAP, or EMD at concentrations of 10 and 50 μg/ml. Cells stimulated with FBS-free ECM supplemented with 0.001 % of acetic acid served as vehicle control. Isolation of total cellular mRNA and transcription into cDNA was performed using the TaqMan Gene Expression Cells-to-CT kit (Ambion/Applied Biosystems, CA, USA) according to manufacturer’s instructions. Real-time PCR was performed on an Applied Biosystems Step One Plus real-time PCR instrument (Applied Biosystems, CA, USA) using TaqMan® gene expression assays with the following ID numbers (all from Applied Biosystems, CA, USA): E-selectin, Hs00174057_m1; ICAM-1, Hs00164932_m1; FLT-1, Hs01052961; KDR-1, Hs00911700_m1; ang-2, Hs01048043_m1; vWF, Hs00169795_m1; GAPDH, Hs99999905_m1). Duplicate PCR reactions were prepared and the point at which the PCR product was first detected above a fixed threshold (termed cycle threshold, C_t_), was determined. Changes in the expression of target genes were calculated using 2^−ΔΔCt^ method, where ΔΔC_t_ = (C_t_^target^ − C_t_^GAPDH^)_sample_ − (C_t_^target^ − C_t_^GAPDH^)_vehicle control_.

### Measurements of cell surface protein expression by flow cytometry

The expression of adhesion molecules ICAM-1 as well as VEGF receptors FLT-1 and KDR on the cell surface of HUVECs was measured by fluorescence flow cytometry [22]. For the measurements of ICAM-1 expression, one part of cells was stained with phycoerythrin-conjugated mouse anti-human ICAM-1 antibody, whereas other part of cells was stained with corresponding isotype control antibody (all eBioscience, San Diego, CA, USA). Surface expression of different proteins was analyzed using a flow cytometer (FACScan, Becton Dickinson, San Jose, CA, USA). Cell counting was limited by 5000 events and the mean fluorescence intensities values were determined for each sample. The expression of ICAM-1 and E-selectin for each sample was quantified using Cell Quest software (Becton Dickinson, San Jose, CA, USA) based on mean fluorescence intensity values of cells stained with ICAM-1 and E-selectin antibodies [23]. Unspecific staining was assessed by measuring cells stained with the isotype control antibody. For the measurements of FLT-1 and KDR expression cells were stained with primary rabbit polyclonal andibodies (all Santa Cruz Biotechnology, Dallas, Texas, USA) and subsequently with secondary goat anti-rabbit antibody conjugated with FITC (eBioscience, San Diego, USA). The percentage of FLT-1- and KDR-positive cells was analyzed by Cell Quest software (Becton Dickinson, San Jose, CA, USA).

### ELISA analysis

Commercially available ELISA kits were used for measurements of vWF (Novateinbio, Woburn, MA, USA) and ang-2 (RayBiotech, Inc., Norcross GA, USA). Before the measurements of vWF and ang-2, samples of conditioned media were diluted with corresponding assay diluent by the ratio 1:10.

### Statistical analysis

The normal distribution of all data was tested with the Kolmogorov-Smirnov test. For normally distributed data, the statistical differences between different groups were analyzed by one-way analysis of variance (ANOVA) for repeated measures followed by post hoc LSD-test. For non-normally distributed data, the statistical differences between groups were analyzed by Friedman test and pairwise comparison was performed using Wilcoxon test for paired variables. All statistical analysis was performed using statistical program SPSS 19.0 (SPSS, Chicago, IL, USA). Data are expressed as mean ± S.E.M. Differences were considered to be statistically significant at *p* < 0.05.

## Results

**Fig. 1:**
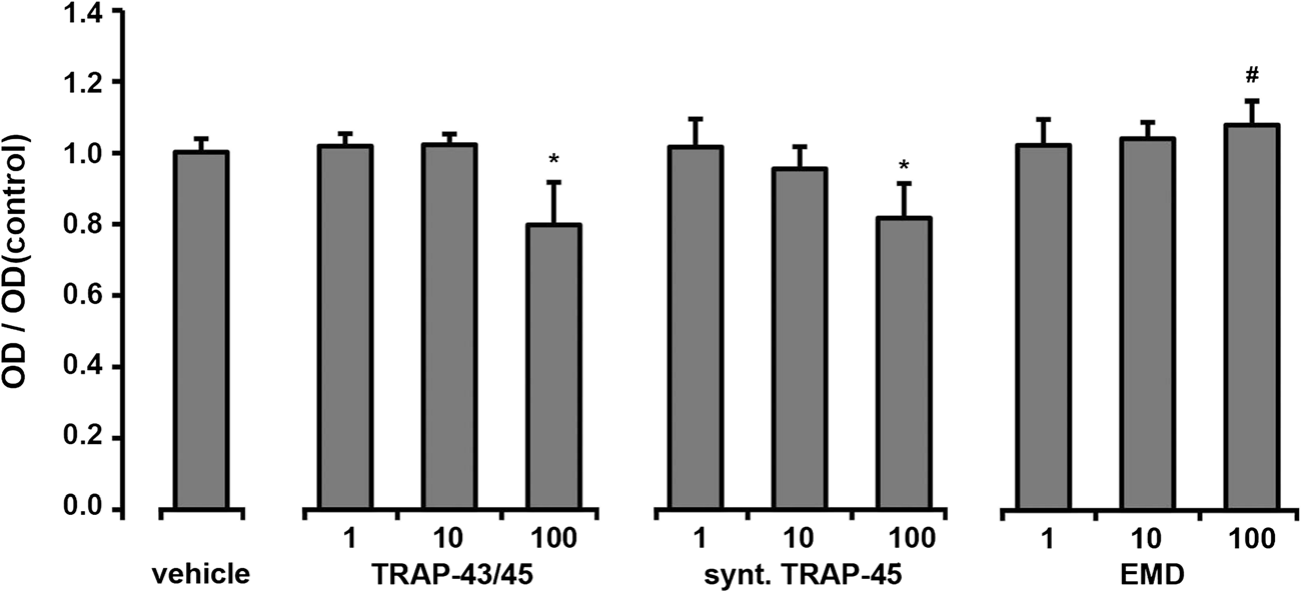
Effect of e-TRAP, synthetic TRAP, and EMD on HUVEC proliferation/viability. Proliferation/viability of HUVECs was measured by MTT assay. HUVECs were treated for 24 h with 1–100 μg/ml e-TRAP, synthetic TRAP, or EMD. Cells treated with 0.001 % of acetic acid were taken as vehicle control. The values of optical density (OD) of the different concentrations were normalized to the average OD value of the control group (=1). Data are presented as mean ± S.E.M. ^#^ *P* < 0.01, significantly higher than the control*.* *P < 0.01, significantly lower compared to the control

Effect of different substances on proliferation/viability of HUVECs measured in MTT assay is shown on the Fig. 1. Proliferation/viability of HUVECs was significantly decreased by both e-TRAP and synthetic TRAP at concentration of 100 μg/ml but was not affected by lower concentrations of these substances (1–10 μg/ml). Treatment of HUVECs with EMD at a concentration of 100 μg/ml increased proliferation/viability significantly compared to lower EMD concentrations.

**Fig. 2:**
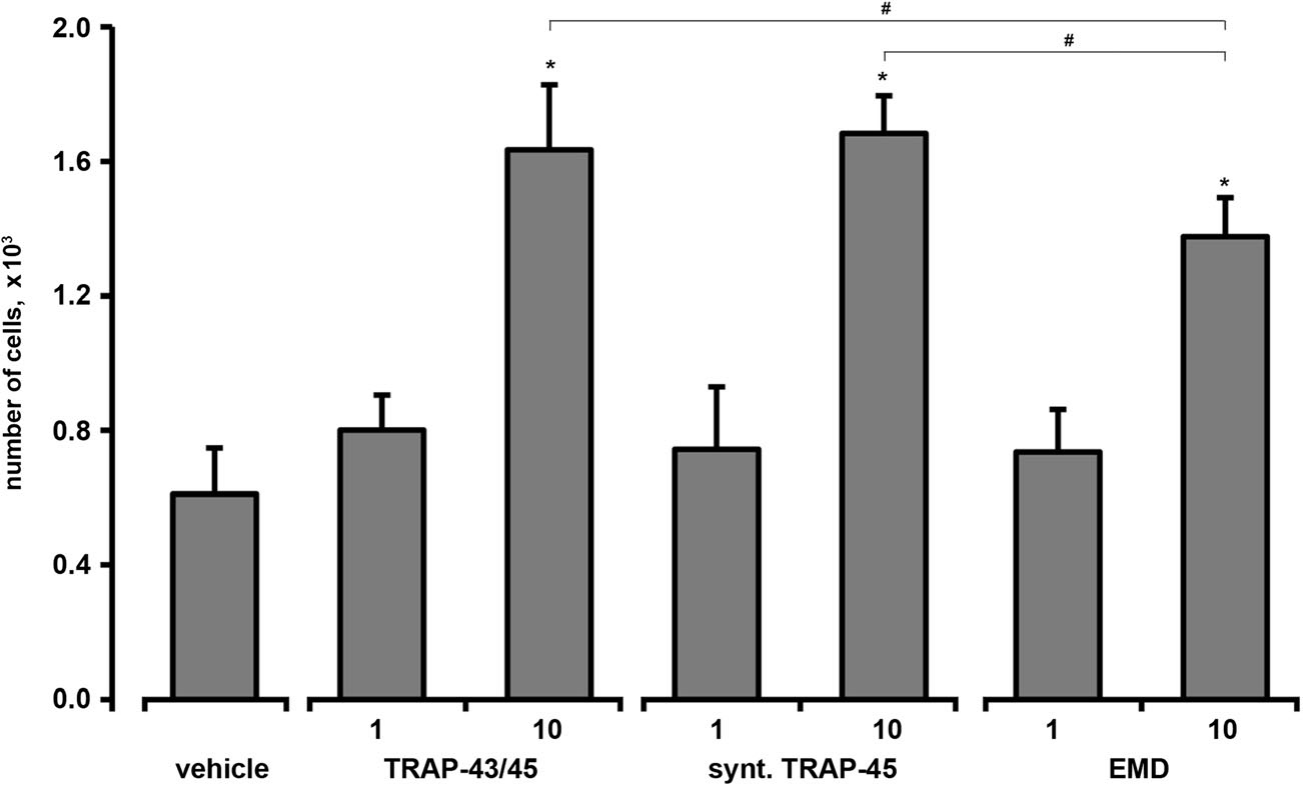
Effect of e-TRAP, synthetic TRAP, and EMD on the migration of HUVECs measured in the microchemotaxis chamber. The number of cells migrated through 8 μm polycarbonate upon stimulation with e-TRAP, synthetic TRAP, or EMD is shown. Stimulation with 0.0001 % of acetic acid served as the control. Data are presented as mean ± S.E.M. *P < 0.01, significantly higher compared to the control. ^#^ *P* < 0.05, significantly different between groups

Migration of HUVECs through 8 μm polycarbonate filter measured in the Boyden chamber was stimulated by all substances at a concentration of 10 μg/ml (Fig. 2). The number of cells migrated through the membrane after stimulation with either e-TRAP or synthetic TRAP was significantly higher than that after stimulation with EMD (*p* < 0.05).

**Fig. 3:**
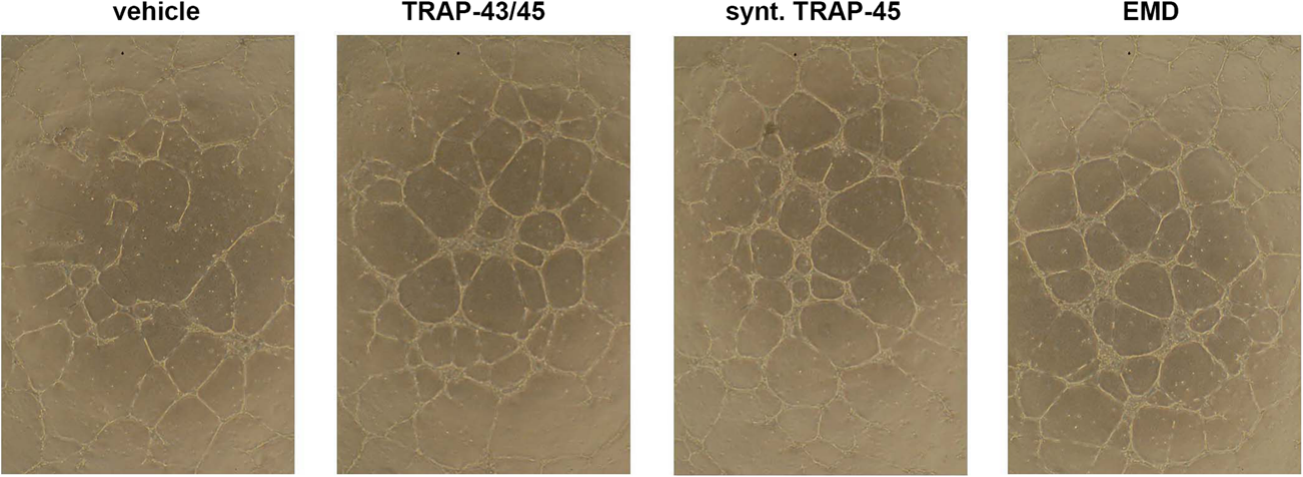
Effect of e-TRAP, synthetic TRAP, and EMD on the formation of angiogenic structures by HUVECs in vitro. Formation of angiogenic structure by HUVECs was detected using angiogenesis kit (Life Technologies) in the presence of e-TRAP, synthetic TRAP, and EMD at a concentration of 10 μg/ml. Cells treated with 0.0001 % acetic acid were used as a vehicle control. Photos are made using light microscope at magnification ×4

Formation of angiogenic structures in vitro was stimulated by e-TRAP, synthetic TRAP, and EMD at a concentration of 10 μg/ml. As can be seen on original photos of angiogenesis assay (Fig. 3), all substances induced more branching points and larger vessel structures. No qualitative differences in the formation of angiogenic structures between different substances were observed.

**Fig. 4:**
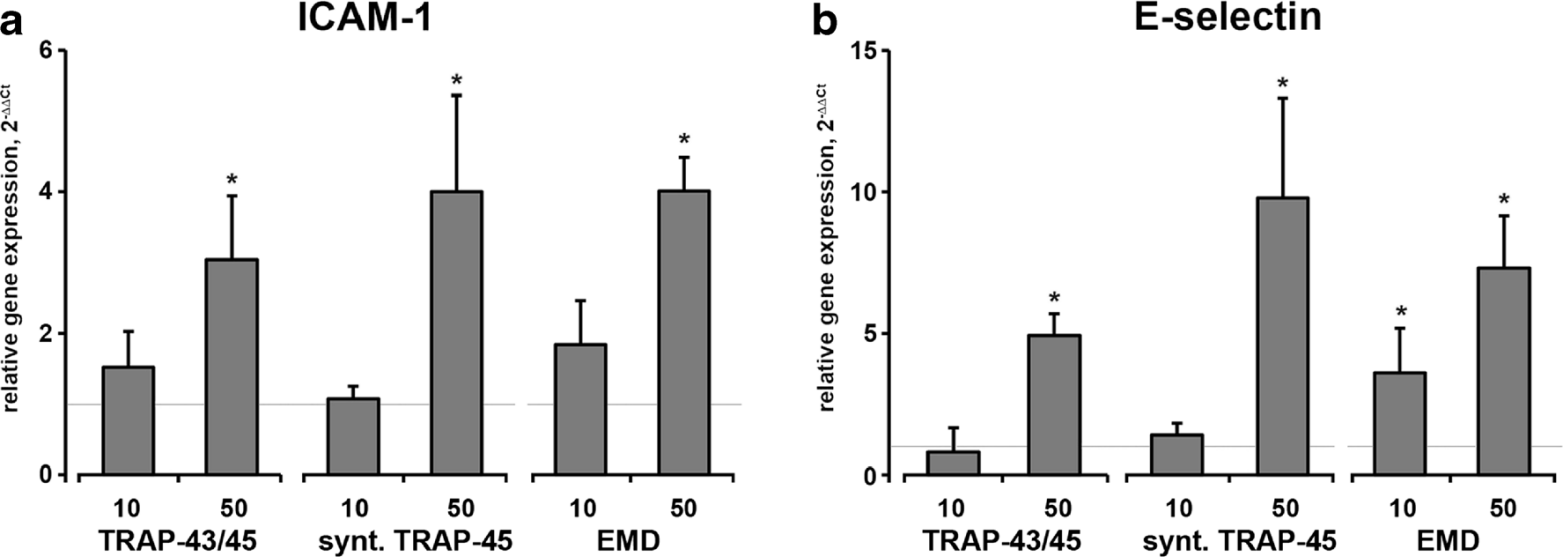
Effect of e-TRAP, synthetic TRAP, and EMD on mRNA expression of adhesion molecules ICAM-1 and E-selectin. Relative expression level of ICAM-1 (**a**) and E-selectin ICAM-1 (**b**) genes upon incubation with e-TRAP, synthetic TRAP, or EMD at concentrations 10 or 50 μg/ml for 24 h. GAPDH was used as endogenous control gene. ECM supplemented with 0.0005 % acetic acid served as vehicle control (=1). Data are presented as mean ± S.E.M. *P < 0.01, significantly higher compared to vehicle control

**Fig. 5:**
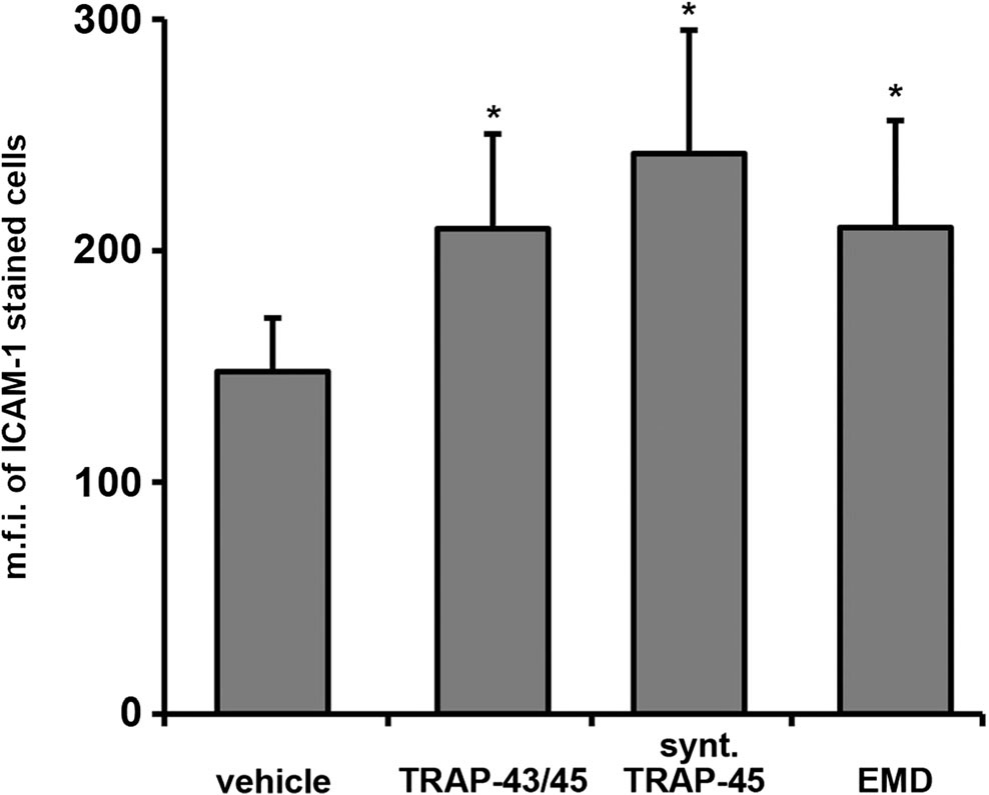
Effect of e-TRAP, synthetic TRAP, and EMD on surface expression of ICAM-1 HUVECs were stimulated with e-TRAP, synthetic TRAP, or EMD at a concentration of 50 μg/ml for 24 h and stained with phycoerythrin-conjugated anti-ICAM-1 antibody. Mean fluorescence intensity (m.f.i.) values of cells stained with ICAM-1 antibodies were corrected for unspecific staining by subtracting the fluorescence of cells stained with the isotype control antibody. Cells treated with 0.0005 % acetic acid served as vehicle control. Data are presented as mean ± S.E.M. *P < 0.01, significantly higher compared to vehicle control

Both e-TRAP and synthetic TRAP at a concentration of 50 μg/ml induced a significant increase in the mRNA expression level of adhesion molecules ICAM-1 and E-selectin in HUVECs (Fig. 4). The effect of both TRAP preparations was not different from that of EMD at similar concentration. Expression of ICAM-1 on the surface of HUVECs was significantly increased by both TRAP preparations at a concentration of 50 μg/ml (Fig. 5). This effect was similar to those of EMD (50 μg/ml). The expression of E-selectin on the surface of HUVECs was not detected by flow cytometry (data not shown).

**Fig. 6:**
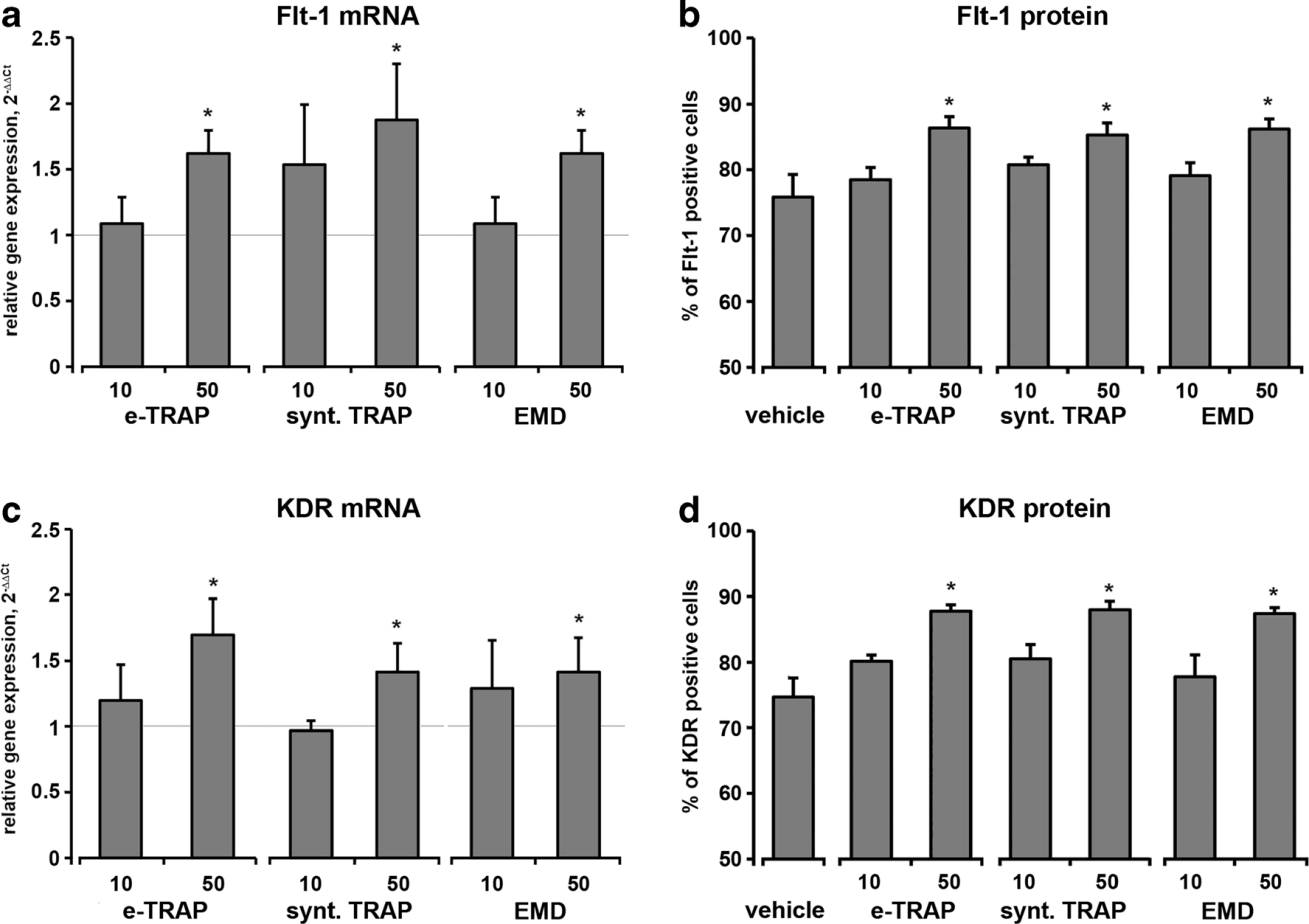
Effect of e-TRAP, synthetic TRAP, or EMD on the expression of VEGF receptors FLT-1 and KDR. **a**, **c** Relative gene expression levels of FLT-1 (**a**) and KDR (**c**) in HUVECs upon incubation with e-TRAP, synthetic TRAP, or EMD at concentrations 10–50 μg/ml for 24 h. GAPDH was used as endogenous control gene. Cells treated with 0.0005 % acetic acid served as vehicle control (=1). **b**, **d** Percentage of FLT-1 and KDR-positive cells measured by flow cytometry after stimulation with the same substances. Data are presented as mean ± S.E.M. *P < 0.01, significantly higher compared to vehicle control

The mRNA expression levels of VEGF receptors FLT-1 and KDR were significantly upregulated by both e-TRAP and synthetic TRAP at a concentration of 50 μg/ml (*p* < 0.05, Fig. 6a, c). This effect was similar to that of EMD. The percentage of FLT-1 and KDR-positive cells was significantly increased by all substances at a concentration of 50 μg/ml (Figs. 6b, d).

**Fig. 7:**
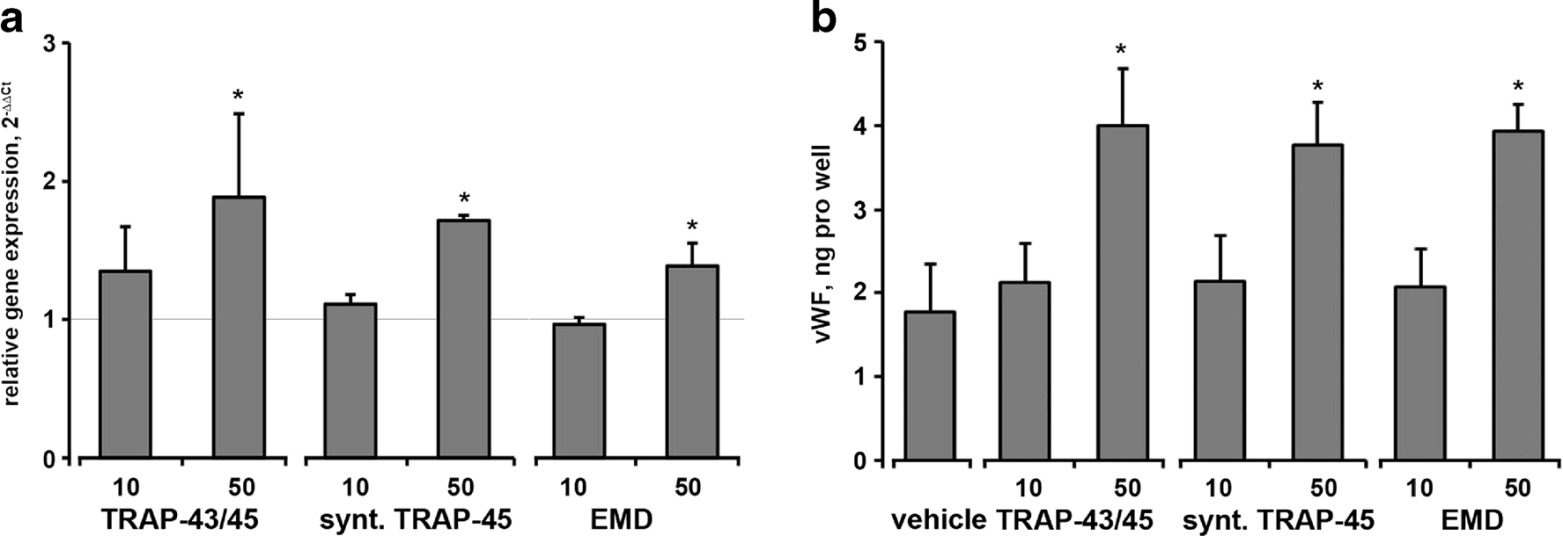
Effect of e-TRAP, synthetic TRAP, and EMD on the expression of vWF in HUVECs. **a** Relative gene expression level of von Willebrand factor upon stimulation with e-TRAP, synthetic TRAP, or EMD at concentrations 10–50 μg/ml for 24 h. GAPDH was used as endogenous control gene. ECM supplemented with 0.0005 % acetic acid served as vehicle control (=1). **c**, **b** The levels of vWF proteins in conditioned media measured by commercially available ELISA. Data are presented as mean ± S.E.M. *P < 0.01, significantly higher compared to vehicle control

The mRNA expression levels of vWF was significantly increased by both e-TRAP and synthetic TRAP (Fig. 7a) at a concentration of 50 μg/ml. Similar increase in the vWF mRNA expression levels in HUVECs was observed upon stimulation with EMD at similar concentration. The content of vWF in the conditioned media was significantly increased upon stimulation with all substances at a concentration of 50 μg/ml (Fig. 7b). No significant difference in the effects of different substances was observed.

**Fig. 8:**
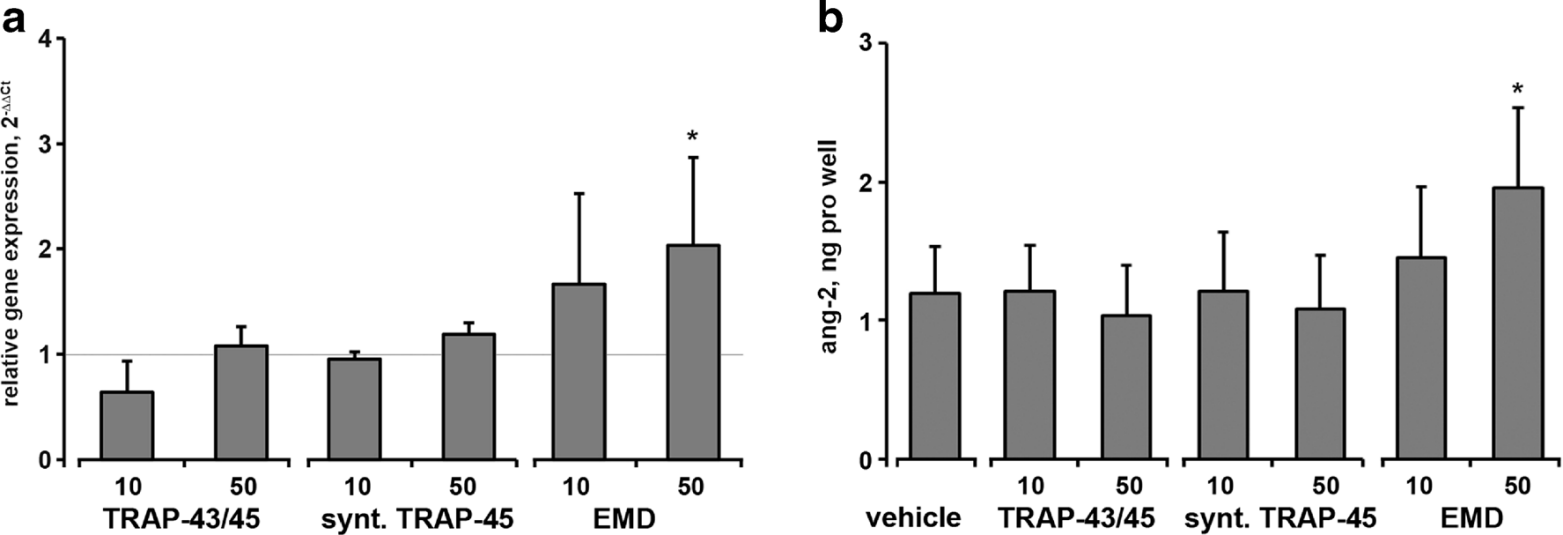
Effect of e-TRAP, synthetic TRAP, and EMD on the expression of ang-2 in HUVECs. **a** Relative gene expression level of angiopoietin-2 upon stimulation with e-TRAP, synthetic TRAP, or EMD at concentrations 10–50 μg/ml for 24 h. GAPDH was used as endogenous control gene. ECM supplemented with 0.0005 % acetic acid served as vehicle control (=1). **c**, **b** The levels of ang-2 proteins in conditioned media measured by commercially available ELISA. Data are presented as mean ± S.E.M. *P < 0.01, significantly higher compared to vehicle control

No significant effect of either e-TRAP or synthetic TRAP (10–50 μg/ml) on the ang-2 mRNA expression levels in HUVECs was observed (Fig. 8a). Similarly, the content of ang-2 in conditioned media was not significantly influenced by these substances (Fig. 8b). In contrast, EMD at a concentration of 50 μg/ml induced significant increase in the mRNA expression levels and protein production of ang-2 by HUVECs.

## Discussion

Commercially EMD-based product Emdogain is successfully used for regeneration of periodontal defects and wound healing since more than 10 years. Process of neovascularization plays an important role in all phases of wound healing: hemostatic clot formation provides a provisional matrix for tissue formation; blood vessels supply nutrients and oxygen and facilitate access of inflammatory cells to the wound [7, 24]. Several studies report that EMD stimulates angiogenesis both in vitro and in vivo; however, the exact EMD components responsible for its angiogenic activity are not exactly known. In the present study, we have investigated the effect of TRAP, a low molecular weight peptide and one component of EMD, on endothelial cells in vitro, in order to explore its potential role in angiogenesis and wound healing.

The proliferation/viability of HUVECs measured by MTT assay was significantly decreased after treatment e-TRAP and synthetic TRAP at concentration of 100 μg/ml. In contrast, EMD itself at similar concentration stimulated the proliferation/viability of HUVECs. MTT assay is based on the measurements of formazan formation by cells mitochondria and therefore is often used as a measure of proliferation of viable cells [25, 26]. Discrepancy in the effects of TRAP and EMD could be accounted by different apoptotic activity of TRAP compared to EMD. Indeed, it is known that EMD at concentrations higher than 100 μg/ml induces apoptosis of endothelial cells [9], which might be related to the presence of some cytotoxic substances in EMD. It is possible that TRAP preparations also possess some cytotoxicity, which appears at lower concentrations compared to EMD. The apoptotic activity of TRAP and EMD might also play an important role in the processes of periodontal tissue regeneration. The process of apoptosis is tightly associated with altered activity of matrix-metalloproteinase (MMP) family proteins [27]. As shown by a previous study, several MMP proteins, namely, MMP-2, MMP-9, and MMP-20 play an important role in the processing and maturation of dental matrix [28]. However, the exact role of MMP proteins in the regenerative effect of TRAP and EMD remains to be elucidated.

Migration of HUVECs measured in the microchemotaxis chamber was strongly stimulated by both TRAP preparations. Moreover, chemotactic ability of TRAP was even higher than that of EMD. Migration of endothelial progenitor cells to the wound cite is a pre-requisite for new vessel formation and therefore is one of the key processes in angiogenesis [29]. This finding is in line with previous observation showing that synthetic TRAP also stimulates migration of human periodontal ligament cells and HUVECs measured in wound healing assay [15].

We further observed that both TRAP preparation stimulated formation of angiogenic structures in vitro by HUVECs. Formation of tubular structures is considered as one of the main features of angiogenic differentiation [30]. Thus, TRAP might confer the ability of EMD to stimulate angiogenesis in vitro observed in some previous studies [9, 10, 31]. This conclusion is also supported by recent study showing that TRAP promotes formation of tubular structures by human periodontal ligament cells [15]. HUVECs are often used as a model of endothelial cells in vitro and are thought to contain a complete hierarchy of endothelial progenitor cells derived from the human umbilical cord [32]. Periodontal ligament cells represent a heterogeneous cell population containing some mesenchymal progenitor cells [33]. Therefore, TRAP seems to stimulate angiogenic differentiation of different types of progenitor cells.

In the present study, we found that both TRAP preparations, similarly to EMD, upregulated the expression of adhesion molecules ICAM-1 and E-selectin in HUVECs. These proteins are usually expressed on endothelial cells surface and mediate the adhesion of inflammatory cells to the endothelium and their subsequent migration to wound sites [34, 35]. As recently reviewed, EMD might affect inflammatory response in different cell types, which contribute to its regenerative ability [36]. Our data suggest that TRAP might have a positive effect in the inflammatory phase of wound healing and might be also involved in other inflammation-related effects of EMD. Furthermore, both TRAP preparations induced an increase in the expression of vWF in HUVEC on both gene and protein levels. Previous report shows that synthetic TRAP might also induce vWF expression on periodontal ligament cells [15]. vWF is involved in the platelet adhesion, and platelet, in turn, might release several factors supporting angiogenesis and wound healing [37, 38].

Both e-TRAP and synthetic TRAP enhanced the expression of VEGF receptors KDR and FLT-1 in HUVECs. VEGF is well-known growth factors playing a central role in angiogenesis and vessel formation [39]. VEGF receptors FLT-1 and KDR are localized on the endothelial cell surface and play an important role in endothelial cells differentiation and organization of blood vessels [40]. Interestingly, previous studies show that EMD upregulate the production of VEGF by different cells of periodontium, such as human gingival and periodontal ligament fibroblasts [10, 41, 42]. Thus, it seems EMD and/or TRAP promote the paracrine interaction between different cell types during the process of wound healing through increased expression of both ligand and receptors. This hypothesis is supported by a recent clinical study, in which the application of Emdogain onto the root surface and into the periodontal pocket resulted in the increase of VEGF expression and microvessel density in gingival tissues [5]. It is known that endothelial cell migration is stimulated by activation KDR by VEGF [43]. This suggests that TRAP might also improve interaction between different cell types and by promoting both VEGF release by resident fibroblasts and VEGF response by endothelial cells.

Interestingly, the effect of both TRAP preparations on the expression of different proteins was similar to that of EMD at similar concentration. This fact is rather surprising and is difficult to explain. However, in a study on mice, the highest in vivo angiogenic activity of 6 kDa protein (presumably TRAP) was observed in amount of 50 ng, which was higher compared to other proteins [6]. A recent study on periodontal ligament cells shows that cell activation cells by TRAP is a complicated process, which involves both interaction with cell surfaces and internalization through endocytosis [44]. Different TRAP activation mechanisms might also result in different concentration dependency. However, the mechanisms of TRAP interaction with endothelial cells are currently unknown and remain to be studied in the future.

No significant effect of any TRAP preparation on the expression of ang-2 was observed. In contrast, EMD induced a significant increase in the expression of ang-2 on both gene and protein levels. ang-2 is involved in vessel maturation and facilitates endothelial cell responsiveness to angiogenic and inflammatory stimuli [45, 46]. This observation means that besides TRAP some other EMD proteins and/or peptides are involved in the angiogenic activity of EMD. This statement is supported by a recent study on mice, in which the effect of the so-called EMD-derived protein pools with different molecular weights on blood vessel formation was investigated in vivo [6]. The authors find the highest angiogenic activity for EMD protein pools containing proteins with a molecular weight of 25, 7, and 5 kDa. In our study, we used TRAP isolated from EMD as well as synthetic TRAP, which did not contain other proteins. The contribution of other EMD components, such as leucine-rich amelogenin peptide (LRAP), shealthin, enamelin, ameloblastin, and tuftelin into angiogenesis remain to be clarified. However, it requires isolation of relatively pure peptides.

Several previous studies investigated the effect of EMD protein fraction with molecular weight about 5 kDa, which is presumably composed by TRAP, on angiogenesis, but their results are partially controversial: some studies suggest an angiogenic activity of 5 kDa EMD protein [6, 15, 31], whereas other study report no effect of 5 kDa EMD protein on the blood vessel formation in the chorioallantoic membrane of the developing chicken eggs [47]. In contrast, synthetic TRAP is shown to stimulate angiogenesis in chicken egg model [15]. This suggests that TRAP preparation method might be an important factor influencing its biological activity.

Summarizing, our data as well as the results of previous studies provided evidences that TRAP possess angiogenic activity. TRAP might be used in the designing of new EMD-based product emphasized on specific aspects of tissue regeneration.
